# Supplementary material for: A Highly Efficient Heterogeneous Catalyst of Bimetal-Organic Frameworks for the Epoxidation of Olefin with H2O2
Source: Molecules. 2020 May 21;25(10):2389. doi: 10.3390/molecules25102389 (PMC7287932; doi:10.3390/molecules25102389)
Supplement: Supplementary file 1 [file molecules-25-02389-s001.pdf]

Supporting information

The charge distribution of atoms on styrene has been calculated by the density functional theory (DFT) method at the B3LYP/ (6-311G (d) level using Gaussian 09 program. The calculated results were as follows:

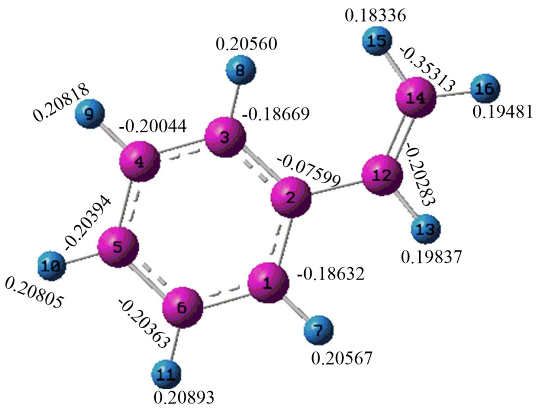

Figure S1 The calculated distribution of NBO charge for styrene

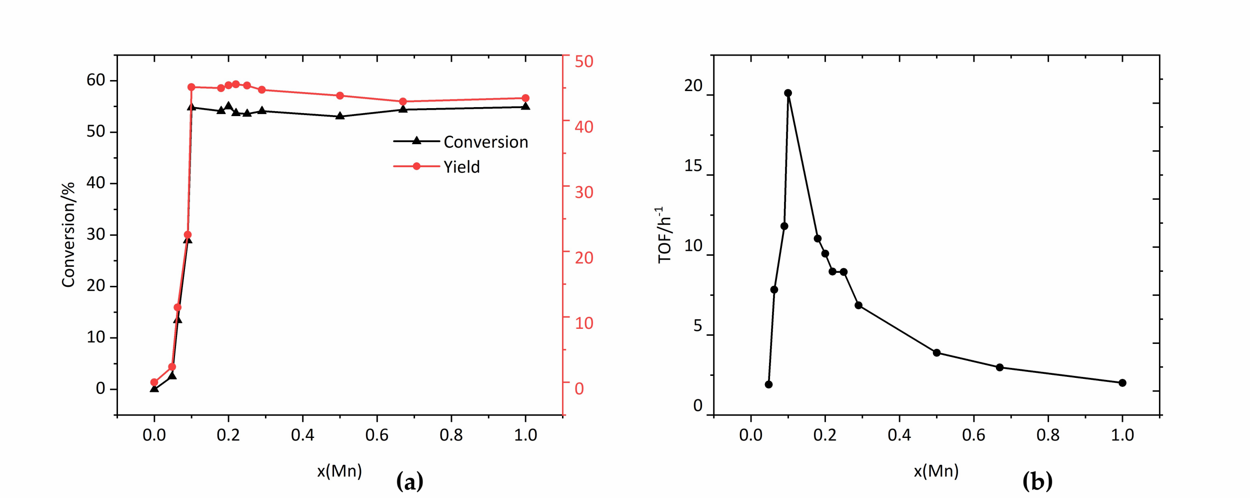

Figure S2. (**a**) Plots of conversion of styrene and the yield of styrene oxide versus a series of Mn_xCu_y-MOFs, 3h. (**b**) The TOF per Mn site with different mole ratio of Mn, 3h.
